# Pilot and feasibility studies: what's the point?

**DOI:** 10.1186/1745-6215-16-S2-P22

**Published:** 2015-11-16

**Authors:** Wei Pek, Martin Ashton-Key, Emma Kirkpatrick, Amanda Young

**Affiliations:** 1 University of Southampton, Southampton, UK; 2 National Institute for Health Research, Evaluation, Trials and Studies Coordinating Centre, Southampton, UK

## Background

The appropriateness of research design and methodology of clinical trials is paramount if we are to succeed in reducing the amount of waste in research. Pilot and feasibility studies serve an important role in determining the most appropriate design and whether the trial will succeed to completion.

## Aims and Objectives

The study will assess the role of pilot and feasibility studies in the design of clinical trials funded by the Health Technology Assessment (HTA) programme.

## Method

There are three phases to the study: 1. Literature review, 2. Review of the ongoing HTA trial portfolio and, 3. Review of the HTA portfolio of published trials to determine the added value of the inclusion of a pilot or feasibility study. A list of HTA trials will be retrieved from three cohorts: completed standalone pilot or feasibility studies; completed and ongoing clinical trials which include an internal pilot or feasibility study; and successful applications in pre-contracting status in the HTA programme.

## Results

The results of the study will still be in development. The number of included trials and proposed checklist/classification system will be presented to determine how pilot and feasibility studies are used to inform the trial design and whether those that include a pilot or feasibility study recruit patients on time and within target.

## Conclusions

The findings from this study will be important in the context of the adding value in research agenda. This is partly due to the lack of existing evidence on the role of pilot and feasibility studies.

